# When death is desired: A case of MAiD & the CL psychiatrist

**DOI:** 10.1017/S1478951524002037

**Published:** 2025-01-21

**Authors:** Amvrine Ganguly, Monique James, Yesne Alici

**Affiliations:** 1 Department of Psychiatry, Mayo Clinic College of Medicine and Science, Rochester, MN, USA; 2 Behavioral Health, Mayo Clinic Health System, South-West Minnesota, Mankato, MN, USA; 3 Department of Psychiatry, Memorial Sloan Kettering Cancer Center, New York, NY, USA; 4 Weill Cornell Medical College, New York, NY, USA

**Keywords:** Consultation-Liaison Psychiatry, Euthanasia, Hospice care, Ketamine, Logotherapy, Meaning Centered Therapy, Medical Aid in dying, Physician Assisted Dying, Palliative Medicine, Psilocybin, PsychoOncology

## Abstract

**Objectives:**

Since physician-assisted dying (PAD) has become a part of the clinical dialogue in the United States (US) and other Western countries, it has spawned controversy in the moral, ethical, and legal realm, with significant cross-country variation. The phenomenon of PAD includes 2 practices: Euthanasia and medical aid in dying (MAiD). Although euthanasia has been allowed in different parts of the world, in the US it is illegal. MAiD has been enacted into law in some jurisdictions. As the practice involves people at the end of life (EOL), often with cancer, and sometimes struggling with psychiatric symptoms; they gain added salience in the field of Consultation-Liaison (CL) Psychiatry in general and Psycho-Oncology in particular.

**Methods:**

The current paper reviews a case where a patient did request for MAiD and successfully carried it through, this case became more salient, as the CL Psychiatry department was intimately linked at various stages of care for the patient.

**Results:**

In describing the case several other aspects of EOL care issues were touched upon, and the various debates as well as treatment modalities, for an individual requesting for medical aid in dying were described.

**Significance of results:**

MAiD will possibly remain a sensitive and controversial topic of discussion across the spectrum of healthcare, and as responsible and compassionate advocates for the patients, clinicians need to engage more with the debate surrounding it and facilitate informed decision making. We believe that the present case will throw light on to this enigmatic practice and help in furthering the dialogue surrounding MAiD.

## Introduction

Ever since physician-assisted dying (PAD) has become a part of the clinical dialogue in the United States (US) and other Western countries, it has spawned controversy in the moral, ethical, and legal realm, with significant cross-country variation (Campbell 2019). The phenomenon of PAD includes 2 practices: Euthanasia and medical aid in dying (MAiD). Although euthanasia has been allowed in the Benelux countries, parts of the Iberian Peninsula, Oceanic countries, and Canada; in the US it is illegal. MAiD has been enacted into law in 11 states and jurisdictions (Buchbinder 2018). As the practice involves people at the end of life (EOL), often with cancer, and sometimes struggling with psychiatric symptoms; they gain added salience in the field of Consultation-Liaison (CL) Psychiatry in general and Psycho-Oncology in particular (Stewart et al. 2018). The current paper will review a case which sheds light on some of these concepts.

### MAiD vs euthanasia

Although euthanasia and MAiD both entail utilization of a potentially life ending medication cocktail, in euthanasia the physician administers the cocktail, whereas in MAiD, it is the patient themselves who administers the medicines (Richardson 2023). This is different from Canada, where virtually all MAID deaths are clinician-administered (i.e. euthanasia) rather than self-administered (Khoshnood et al. 2018). Of note, provider and facility participation is voluntary for MAiD in the US. As previously discussed, euthanasia has been legal in other countries; however, it is still not legal in the US. MAiD is the only form of PAD allowed in these US jurisdictions.

### The case

Ms. X is a 78-year-old woman with no significant past psychiatric history prior to her diagnosis of urothelial cancer. The patient underwent extensive cancer resection surgery and was placed on a novel immunotherapeutic regimen. Although the cancerous process halted after the resection surgeries, the resultant side effects from the immunotherapy were debilitating, including several somatic and autonomic symptoms (e.g., nausea, dizziness, malaise, difficulty focusing, headaches, blurry vision, severe neuropathy, low-grade fever.) The patient came to the hospital in light of these worsening symptoms. It was at that point the psychiatry CL service was consulted on the patient; the primary consult request was made for the patient’s perceived demoralized status. The inpatient CL team assessed the patient and recommended outpatient psychiatry follow up after discharge. It was during this hospital-based evaluation that the patient first discussed wanting to pursue MAiD.

In her outpatient assessments, the patient endorsed her frustration from the neurological side effects, particularly numbness and the occasional inability to move, which emanated from the neuropathy resulting from the chemotherapeutic regimen. She strongly expressed her thoughts of optimizing her EOL care – for however long – so she could have the highest quality of life for the longest time possible. The patient was consistent and clear about her intention of ending life on her own terms if the symptoms associated with her cancer or non-abating side effects of the cancer treatment progressed and reached her own preset threshold for tolerance. The patient’s family was aware, in agreement, supportive and protective of her decision. The patient also denied any imminent plan/intent to harm or kill herself and was future oriented. There was no significant psychiatric history to suggest that the patient had been suffering from any form of mania, psychosis, panic symptoms, depression, PTSD, or substance use disorder in the past which may contribute to any form of decompensation in her mental state.

In terms of psychosocial history, she was a college professor. She lived by herself in her own apartment. She reported losing her husband 10 years prior from cancer and endorsed the suffering he endured as one of the strongest reasons for her to choose her own time of death.

As the patient and family expressed interest in some form of PAD, the MAiD process was extensively discussed. But, as MAiD was not legal and unavailable in her state of residence, they decided to take their time in further proceeding with it. The patient and her family at this point of time, were unsure about choosing the location to undergo the MaiD procedure, and as almost all the states where MaiD has been legalized, statutory language limits the use of the practice to instate residents, considerable planning was required for it. The patient also dropped out of active follow-up visits with outpatient psychiatry, though accepted telephone-only check-ins for her well-being.

Two months after her last follow up, the patient presented to the hospital for a novel medication trial for her neurological side effects. It was only late in her admission day that she admitted to overdosing on opioids she was prescribed for cancer-related pain. The patient endorsed taking a bottle of morphine pills, however the number of tablets were never specified. As her clinical condition worsened, she was transferred to the ICU and received multiple doses of Narcan to counter the opioid toxicity. Psychiatry was consulted again for assessment of her mental state. The patient presented with delirium on her initial assessment and was unable to hold a conversation. During her ICU stay and later transfer on to the general floors, she was placed on suicide precautions as per hospital policy. She was followed up on a regular basis by the inpatient CL Psychiatry team for daily evaluations.

On subsequent assessments the patient cleared up in terms of her mental status. She expressed remorse about overdosing on the opioids and stated she made an error in trying to take her own life by this method, which she described as “crude and undignified.” The patient, though, was steadfast in her refusal that there were any prevailing mood symptoms which would have spurred suicidal ideation or thought. Instead, she painted this as an impulsive reactionary attempt at self-harm, in response to interpersonal distress in her relationship with her family. Extensive collateral history was obtained from her 2 daughters. They reported the patient had a baseline temperament of low adaptability and high mood intensity and thought this attempt was the result of a recent interpersonal struggle with 1 daughter. Both daughters, however, denied the presence of any signs or symptoms suggestive of depression in the weeks leading up to this event.

The patient made an uneventful medical recovery in the ICU and then on the general medical floors. She consistently denied any imminent suicidal ideation, impulse, or thoughts and suicide precautions were discontinued. The patient was started on a trial of antidepressant (Sertraline) to counterbalance her mood-based impulsivity. Throughout her hospitalization, she maintained her initial stance of choosing how and when to terminate her life. She was extensively counseled on the requirements, eligibility, and procedure of the MAiD process. A palliative care consultation was completed to ensure excellent symptom control. Her daughters were co participants in her safety planning. Furthermore, the Psychiatry CL team thoroughly liaised with the other specialties of her inpatient team in confirming her capacity for medical decision making, reassuring the team there was no acute psychosis or acute depression, and to continue to assess for existential distress as well as keeping in consideration the patient’s decision to pursue MAiD. Incidentally the National Comprehensive Cancer Network (NCCN) designated cancer center in which she was being treated did not directly participate in MAiD and did have institutional standards to navigate these requests, as they included coordination of care at different levels in the inpatient and outpatient level. The patient would often start bedside interviews with “I don’t want to talk about it, you’re not changing my mind” to establish her autonomy.

The patient was subsequently discharged with an appointment in outpatient follow-up with Psychiatry. Interestingly, in the outpatient follow-up visits the patient was steadfast in her maintained need of MAiD and showed a tendency toward provocative statements surrounding her wish of voluntary death. During the span of writing this article, she continued to be followed up by oncology and psychotherapeutic services, and finally pursued MAiD at home.

## Themes

The above-described case brought forth several features of this already controversial and sensitive topic into the light. Although this specific patient was fortunately insightful enough to bring herself into the hospital after her apparently impulsive suicidal attempt (though for a different medical reason), her condition closely mimics the clinical condition of several others (elderly population with a terminal illness) who might be opting for physician-assisted death and opens up important questions for CL Psychiatry, particularly for physicians who are operating in Psycho-Oncology settings.

### Depression and MAiD

Depressive symptoms are common at the EOL, and there is longstanding concern that it may affect terminally ill patients’ decisions to request PAD. However, it is important for clinicians to determine if the role of depression is playing a part in the patient who requests PAD. To begin with, PAD has historically been an extremely delicate and controversial topic, and has generated considerable reflection, both within the US and internationally. Countries like Belgium, The Netherlands, Luxembourg, and Switzerland have allowed people living with mental disorders to access some forms of MAiD for years and more recently, Spain has passed MAiD laws making people living with mental disorders eligible for MAiD under certain conditions. In Canada, after much deliberation the enactment of MAiD based solely on mental health disorders has been indefinitely postponed. In the US, as MAiD is the only legal form of PAD, it is limited to self-administration of a drug by those with terminal medical illnesses. Psychiatric/mental disorders, which might be treatment refractory, are still kept out of the ambit of MAiD. Consequently, when a patient with depressive symptoms makes a PAD request, the clinician’s role becomes challenging, as depression may affect a patient’s decision making and judgment (Blank et al. 2001; Ganzini et al. 1994). Whether this “drives their desire for PAD,” and how to determine this role of depression is not always clear. For this patient, although she denied any symptoms of depression and her family was steadfast in denying any signs of the same, her self-harming attempt, when placed in the context of her impulsive temperament, raises the question on how much her decision of undergoing MAiD fueled this apparent impulsive attempt. Aside from a diagnosis of clinical depression, a patient suffering from a rapidly worsening incurable condition, may have several other psychological reactions to their clinical state including demoralization, death anxiety, or employing maladjusted coping mechanisms, which might play into a wish for hastening death. Also, this particular case revealed that follow-up with psychiatric care might be tenuous in a subcategory of patients. The wider debate circumscribing this episode is the difference between suicide and MAiD.

### MAiD & suicide: phenomenological difference

The website of Death with Dignity, one of the largest organizations seeking to legalize medically assisted dying in the US, offers an explanation to differentiate between the two, where they claim that in MAiD/PAD “the patient’s primary objective is not to end an otherwise open-ended span of life, but to find dignity in an already impending exit from this world. They’re participating in an act to shorten the agony of their final hours, not killing themselves; cancer (or another common underlying condition) is killing them.” In short, in choosing MAID, one is not deciding on “if” one dies but rather “how” one dies. There is of course intense and sometimes acrimonious debate based on morality, capacity, and motivation for such a death. One central distinguishing point between the two, is the desire for death, which in a case of suicide, emerging from an underlying psychiatric disorder (mostly depression), might be a core feature of the diagnostic criteria, whereas in MAiD, the death wish is more reactive, one which is emerging from a fine interplay/balancing between degree of hope and irremediability of suffering (Friesen 2020). Life looked through the lenses of depression can distort reality, whereas in a terminal illness, eventual death is the reality. Although clinical depression has multimodal treatment strategies, the philosophical argument could be made, that even in suicide the person is reacting to an interminable mental condition of depression, and is hence reacting to “psychache,” the psychical equivalent of somatic pain and suffering. Given, that depression is a frequent accompaniment in terminal and advanced cancer symptoms, the delineation and in that respect, assessing for capacity gains paramount importance.

### Novel treatment of depression

As is evident from the case presented and from the ensuing discussion, it is sometimes difficult for clinicians to determine the role of depression in a patient’s PAD request. Most traditional treatments for depression require a lengthy trial period (as well as have potential systemic side effects in medically – compromised patients). There has been a recent emergence of what might be called rapid-response treatments (RRT) for depression with a potential to elicit a meaningful response even in patients with short life expectancies (Berens and Kim 2022). This may point to a renewed need to consider their implications for the practice and policy of PAD. Ketamine (Goldman et al. 2019), Psilocybin (Grob et al. 2022), and iTBS (Cole et al. 2022) work by novel mechanisms, with the goal of alleviating the depression and clarifying the thought process. If the patient does respond to the treatment, it reduces the potential for erroneous capacity assessments – both false positives of incapacity leading to deprivation of a person’s legal right to receive PAD, and false negatives that lead to premature deaths and deprivation of potentially meaningful last days in people’s lives. In this regard mention must be made, about the landmark study, conducted in Canada a few years ago, where a small cohort of patients with PAD requests, received Ketamine, and showed uniform response in terms of resolution of their depressive symptoms. Ketamine administration being labeled as the “litmus” test clarified and distilled their decision-making capacity. Indeed, in the US where assisted dying is not permitted for depression alone novel interventions like Ketamine can better inform the current legislative debates on this issue (Rosenblat and Li 2021). However, this is largely unchartered territory, with a number of potential ethical, moral, and medical implications (Hermann et al. 2016). For example, what happens if an individual with depressive symptoms is requesting PAD, but refuses RRT? The other question to consider would be the accessibility of such treatments (Puyat et al. 2016). Moreover, there is still a paucity of data on this subject matter, and case reports or series have been limited.

### Looking outside medicine: Role of MCP

In 1959 Victor Frankl wrote the book Man’s Search for Meaning (Frankl 1967) in which he proposed that life in itself never ceases to have meaning, even during the most dark and insufferable times that one can go through. Keeping Victor Frankl’s principle of logotherapy at its core, (Breitbart 2016) came up with a unique therapeutic protocol based on their research conducted in Memorial Sloan Kettering, NY. Termed meaning-centered psychtherapy, this specific form of psychotherapy sees the patients’ lives as a form of living legacy, where the past is reflected upon, the present is examined, and the future contemplated. Although no therapeutic trial has been conducted on the specific population opting for MAiD, evidently with people at the end of their life, this specific form of therapy which is geared toward finding the meaning in the lived life, may offer an alternate to the newer medical protocols in addition to offering closure.

### Assessment of irremediability of suffering

PAD has different implications for clinicians, depending on their jurisdiction’s irremediability requirement. In the Netherlands, for example, a physician must conclude “together with the patient that there is no reasonable alternative” to PAD, whereas Canadian law requires a “grievous and irremediable medical condition” that must cause patients “enduring physical or psychological suffering that is intolerable to them and that cannot be relieved under conditions that they consider acceptable” (Nicol and Tiedemann 2015). Whereas the patients’ subjective judgment acts as the driving basis for determining tolerability/intolerability, irremediability is a quality determined by clinicians, i.e., stage 4 cancer is irremediable. In these scenarios, relying heavily on the patient’s perspective may be problematic when their depressive symptoms may be driving their perceptions of what is “intolerable to them” and what treatments “they consider acceptable.” New Jersey (NJ) is one of the 11 states of the USA, where MAiD is legal for the terminally ill patients who are mentally capable, can communicate their choice, and can physically self-administer the lethal combination of medicines (Downie et al. 2022). Significantly, the patient in this case, had contemplated the idea of going to the state of NJ for MAiD. It is to be remembered, however, that no psychiatric assessment is necessarily mandated as per the NJ guidelines, hence leaving the irremediability of suffering concept as to be somewhat vague and nebulous. As for the case in point, the patient repeatedly expressed her wish of ending life, when her “condition” got irreversibly worse. Thus, the concept of “irreversibly worse” is left up to the patient to develop and craft. It also should be clarified, that in the US, every state that has legalized medically assisted dying to date requires that natural death is foreseeable within the next 6 months, hence the “terminally ill” are being defined based on a foreseeable period of life expectancy as opposed to intolerable suffering, in Canada however this criterion is more loosely based as reasonably foreseeable natural death (Downie and Scallion 2018), which does not have a numerically defined cut-off point. Based on whichever side one is on the MAiD debate, this might be labeled as a strength or weakness of the legal system.

### Role of palliative care

Though superficially, the concept of MAiD & palliative care might seem to be at cross-purposes, an in-depth examination in fact reveals that palliative care and MAID do not have to be mutually exclusive and are often complementary and synergistic for patients and families. Unfortunately, the gap in access to palliative care still is a daunting task for the terminally ill patients (with and without MAiD) to avail of this service, how far this unavailability is contributing toward the decision for MAiD has not been studied yet in most US jurisdictions. Hospice care is commonly provided by an interdisciplinary team involving a wide range of care providers including physicians, regulated nurses, care aides, social workers, and grief support counsellors as well as volunteers and administrative staff. Studies in Oregon, Washington, Belgium, and Canada where the majority of patients who received euthanasia or PAD had received palliative care (Emanuel et al. 2016) (Li et al. 2017) show that for some individuals, the desire for MAiD persists despite receiving palliative care. This may be explained by a multitude of reasons, including a sense of fatality while facing an incurable condition, lack of autonomy, desire for control, loss of dignity, and an inability to enjoy activities of daily living (Wilson et al. 2007). Patients should be educated that palliative care and MAID are not mutually exclusive options at the EOL and pursuing MAID should not preclude attempts to control symptoms or improve quality of life. Although some patients might be well informed of palliative care and still refuse it, others might not be fully informed and require a conversation of how palliative care can help them, a conversation that is currently not well documented in the MAID assessments.

## Conclusion and future direction

MAiD has and will possibly remain a sensitive and controversial topic of discussion across the spectrum of healthcare, and as clinicians advocating for the rights of our patients, there is an obligation for us to take part in this growing conversation to assess and remedy the patient suffering, regardless of our stance on this debate. As technology and its skillful deployment bring the world closer, newer techniques are being discussed and experimented with to bring forth an ending to suffering lives. Some proponents would call this “merciful,” while some critics label these as further corporatization of healthcare at the expense of a human life. Upcoming concepts like “Death Tourism” (Shondell Miller and Gonzalez 2013) and “Death-Doulas” (Rawlings et al. 2019) could be seen as blessings or curses, based on where one’s position is on the morality and ethics of life/death duality. The patient case we kept at the center-point of our discussion, touched upon topics of depression, existential distress and how that may induce patients into relative impulsive acts with low levels of baseline frustration tolerance, and in the wider perspective laid emphasis to the importance of CL Psychiatry where careful assessment of such patients is crucial. At this point of time, there is still a large room for discussion regarding the autonomy or self-directive principle inherent in the practice of MAiD, which we hope will be elaborated on in future studies and writings.
